# Intermediate hosts of the trematode *Collyriclum faba* (Plagiochiida: Collyriclidae) identified by an integrated morphological and genetic approach

**DOI:** 10.1186/s13071-015-0646-3

**Published:** 2015-02-08

**Authors:** Petr Heneberg, Anna Faltýnková, Jiří Bizos, Milena Malá, Juraj Žiak, Ivan Literák

**Affiliations:** Third Faculty of Medicine, Charles University in Prague, Ruská 87, CZ-100 00 Prague, Czech Republic; Institute of Parasitology, Biology Centre ASCR, Branišovská 31, CZ-370 05 České Budějovice, Czech Republic; Administration of the Veľká Fatra National Park, P. O. Hviezdoslavova 38, SK-036 01 Martin, Slovakia; Department of Biology and Wildlife Diseases, Faculty of Veterinary Hygiene and Ecology, University of Veterinary and Pharmaceutical Sciences Brno, Palackého tř. 1/3, CZ-612 42 Brno, Czech Republic

**Keywords:** Cercariae, DNA analysis, Fluke, Host-parasite interaction, Hydrobiidae, Life cycle, Littorinimorpha

## Abstract

**Background:**

The cutaneous monostome trematode *Collyriclum faba* (Bremser in Schmalz, 1831) is a bird parasite with a hitherto unknown life cycle and highly focal occurrence across the Holarctic and Neotropic ecozones.

**Methods:**

Representative specimens of benthic organisms were sampled at multiple sites and dates within the known foci of *C. faba* occurrence in Slovakia. A combined approach involving detailed morphological examination and sequencing of two independent DNA loci was used for their analysis.

**Results:**

We elucidated the complete life cycle of *C. faba,* which we determined to include the aquatic gastropod mollusk *Bythinella austriaca* (Frauenfeld, 1857) as the first intermediate host, the mayflies of the family Heptageniidae, *Ecdyonurus venosus* (Fabricius, 1775) and *Rhithrogena picteti* Sowa, 1971 x *iridina* (Kolenati, 1839), as the second intermediate hosts, and birds (primarily but not exclusively passeriform birds) as the definitive hosts. *Bythinella austriaca* occurs focally in the springs of tributaries of the Danube in the Alpine-Carpathian region. The restricted distribution of *B. austriaca* explains the highly focal distribution of *C. faba* noticed previously in spite of the broad distribution of its second intermediate and definitive host species. Utilization of both larval and adult Ephemeroptera spp. as the second intermediate hosts explains the known spectrum of the definitive host species, with the highest prevalence in species feeding on larvae of Ephemeroptera, such as *Cinclus cinclus* (Linnaeus, 1758) and *Motacilla cinerea* Tunstall, 1771, or adults of Ephemeroptera, such as *Sylvia atricapilla* (Linnaeus, 1758) and *Regulus regulus* (Linnaeus, 1758). In this study, we also determine the prevalence and DNA sequences of other immature trematode specimens found in the examined benthic organisms (particularly the families Microphallidae, Troglotrematidae and Nanophyetidae and *Euryhelmis zelleri* Grabda-Kazubska, 1980, Heterophyidae), and describe cercariae of *C. faba*.

**Conclusions:**

We determined the full life cycle of the Central European populations of *C. faba*. We speculate that other species of *Bythinella* and the closely related genus *Amnicola* may serve as first intermediate hosts in other parts of the distribution range of *C. faba*. Similarly, other Ephemeroptera of the family Heptageniidae may serve as the second intermediate hosts of *C. faba* in the Americas.

**Electronic supplementary material:**

The online version of this article (doi:10.1186/s13071-015-0646-3) contains supplementary material, which is available to authorized users.

## Background

The cutaneous monostome trematode *Collyriclum faba* (Bremser in Schmalz 1831) is a digenetic flatworm with a hitherto unknown life cycle. The definitive hosts include a broad range of birds; *Erithacus rubecula* (Linnaeus, 1758), *Sylvia atricapilla* (Linnaeus, 1758), *Regulus regulus* (Linnaeus, 1758), *Fringilla coelebs* Linnaeus, 1758 and *Corvus brachyrhynchos* Brehm, 1822 have been reported the most frequently parasitized species [1]. It was assumed that the first intermediate host is possibly an aquatic snail [2], and that the second intermediate host is an insect (possibly a dragonfly [3,4] or a mayfly [2]). However, the life cycle remained enigmatic. Already in the 1940s, D. S. Farner and B. B. Morgan collected several hundreds of *Planorbis* sp. and *Stagnicola emarginata* (Say, 1821) and checked these snails for the presence of *C. faba* but without success [2]. Later, A. Faltýnková and I. Literák attempted to find cercariae of *C. faba* within 3802 *Bythinella austriaca* [5]. At Slovakian and Czech sites of focal occurrence of *C. faba*, this benthic snail species is highly dominant (or the only species), and occurs characteristically in springs and small streams. Although A. Faltýnková and I. Literák found 12 morphospecies of trematode cercariae, they did not apply any molecular analyses and classified all the specimens based on morphology as belonging to the families Troglotrematidae, Nanophyetidae, Allocreadiidae, Lecithodendriidae, Microphallidae and the superfamily Opisthorchioidea [5].

In its definitive hosts, the passeriform and sporadically some other birds, the adults of *C. faba* occur in pairs in subcutaneous cysts. The cysts are able to develop in less than 13–19 days [6] and may heal spontaneously after a period of several weeks or months. The abundance of *C. faba* is highly site-specific and the infections prevalently occur in several bird host species only, which is probably related to the restricted distribution range of intermediate hosts and to the spectrum of food ingested by the definitive hosts. In endemic areas, the cysts of *C. faba* were found in 20% of *Motacilla cinerea* Tunstall, 1771 in the Central European Carpathians [6] but, in contrary, only 0.005% of *Riparia riparia* (Linnaeus, 1758) in Central Europe were infected with *C. faba* [7]. Most of the birds only harbour up to four cysts and do not display any major health problems [8] but heavily parasitized birds with over 50 cysts display anemia, emaciation, defecating problems and death [6,9]. In combination with viral infections, including West Nile virus and avian poxvirus, the disease severity may be further strengthened [10].

Only limited information can be retrieved from studies of species closely related to *C. faba*. Some classification systems suggest the two *Cortrema* spp. as members of the family Collyriclidae but the recently published 28S rDNA sequence of *Cortrema magnicaudata* (Bykhovskaya-Pavlovskaya, 1950) suggests that they form a distinct family Cortrematidae [11]. The life cycle of *Collyricloides massanae* Vaucher, 1969, the only other member of the family Collyriclidae, is little known. This morphologically very similar species occurs in intestinal cysts of small rodents and birds. Its intermediate hosts are unknown and its species status is questioned [12]. The family Collyriclidae was previously classified as a member of the super family Gorgoderoidea, but DNA sequencing of *C. faba* revealed that it segregates with members of the superfamily Microphalloidea, particularly with the family Prosthogonimidae [1]. T. H. Cribb et al. reported that the second intermediate hosts of the superfamily Microphalloidea belong to Annelida (two trematode species), Arthropoda (50 species), Mollusca (12 species) and Vertebrata (17 species) [13]. Generally, cercariae of the Microphalloidea are simple-tailed, have a stylet, emerge from gastropods and penetrate arthropod second intermediate hosts; sporocysts prevail over rediae in most of the Microphalloidea species. The stylet is considered an important adaptation allowing the infection of arthropods by penetrating their cuticle or arthrodial membranes. Metacercariae are formed most frequently in the arthropods but may occur in almost any animal. In some Microphallidae, the cercariae may encyst within first intermediate hosts. Generally, the life cycle is considered a poor predictor for classification of the respective taxon into the superfamily Microphalloidea. It is unclear, whether and to what extent these variations are genuine or whether they reflect weaknesses in the current classification and phylogeny [13]. Importantly, the microphallid species exclusively utilize prosobranch mollusks as their first intermediate hosts. Microphallids also use birds and mammals as their definitive hosts. In constrast, gorgoderids utilize bivalves as their first intermediate hosts, and fish and reptiles as their definitive hosts [14]. DNA recombination involving chi-like sequences occurs in parthenites of *C. faba*, and these sequences have been identified in the family Microphallidae as well, whereas they have not been reported in any members of the family Gorgoderidae [15,16].

The diversity in digenean life cycles and the enormous number of known exceptions [14] prevents precise prediction of the life cycle in species with missing data, particularly in those for which any family-specific evidence is absent. The complicated life cycles of trematodes make them highly vulnerable to changes in the intensively cultivated cultural landscape [17]. The absence of intermediate hosts at the sites of occurrence of their definitive hosts (and vice versa) is typically associated with the processes of urbanization, synanthropization and even with widespread inconspicuous eutrophication, often leading to the regional extinction of a particular digenean species [18-22]. However, most of the changes in the abundance and diversity of trematodes are still hidden to our eyes due to the limited amount of population-scale studies performed and the lack of knowledge of the life cycles of the many trematode species.

Using a combined genetic and morphological approach, we elucidate the life cycle of *C. faba* and report the morphological features of the cercariae of *C. faba* together with notes on its occurrence in first and second intermediate hosts. We also focus on the prevalence of *C. faba* in its intermediate hosts and perform genetic profiling of *C. faba* and related species present in the examined putative intermediate hosts taken from a benthos of mountain springs and streams at a site of endemic focal occurrence of *C. faba* in Slovakia.

## Methods

### Sampling

For DNA analyses, we used specimens of prevalently benthic invertebrates collected from two sites of known focal origin of *C. faba* [6], in the Veľká Fatra Mts. and in Bukovské Vrchy Mts., Slovakia in years 2011–2013 (Table 1). During the first two sampling events, the specimens were collected quantitatively, representing the whole spectrum of benthic invertebrates available onsite. With respect to the predicted life cycle of *C. faba*, it was notable that at all the examined sampling sites, only a single benthic snail species (*Bythinella austriaca*) occurred. As a control, we utilized adult *C. faba* specimens obtained previously from *Saxicola rubetra*, *Hirundo rustica*, *Erithacus rubecula*, *Sylvia atricapilla* and *Regulus regulus* [1,16].

**Table 1:** **Sampling sites and dates**

**Sampling site**	**Sampling site, coordinates, date**
1	Gaderský stream, Veľká Fatra Mts., 48°53′N, 19°03′E, 930 m a.s.l., 10-11-Jun-2011
2	Gaderský stream, Veľká Fatra Mts., 48°53′N, 19°03′E, 930 m a.s.l., 3-Aug-2012
3	Gaderský stream, Veľká Fatra Mts., 48°53′N, 19°03′E, 930 m a.s.l., 20-Jun-2013
4	Gaderský stream, Veľká Fatra Mts., 48°53′N, 19°03′E, 930 m a.s.l., 16-Nov-2013
5	Gaderský stream, Veľká Fatra Mts., 48°53′N, 19°03′E, 1000 m a.s.l., 16-Nov-2013
6	Bukovské Vrchy Mts., 49°04′N, 22°23-25′E, 530–620 m a.s.l., 21-24-Jun-2011

The research did not involve collection of any vertebrates or any regulated invertebrates. The collection of benthic invertebrates was permitted by the Administrations of the Veľká Fatra National Park.

### Handling of invertebrates for morphological examination

Snails of *Bythinella austriaca* were individually placed in small plastic containers with dechlorinated tap water and were left overnight at 5°C to allow the cercariae to emerge; other snails were kept together on wet leaves in the dark at 5°C. Afterwards, all snails were dissected under a stereomicroscope to identify cercariae that did not emerge, and other intramolluscan stages, such as sporocysts and rediae.

The collected aquatic insects and crustaceans were fixed in 96% ethanol and dissected under a stereomicroscope. In 2013, the arthropods were dissected live.

Live cercariae and metacercariae were observed under an Olympus BX51 light microscope. Metacercariae were excysted with slight pressure on the cover slide or with a needle. To document the morphology, a series of digital photographs for each isolate were taken using a digital camera attached to the microscope. The measurements (in μm) for live and fixed metacercariae were taken from digital images with the aid of QuickPHOTO CAMERA 2.3 image analysis software. Live cercariae were identified according to Faltýnková et al. [5] and Yamaguti [23]. After identification, representative specimens of cercariae, sporocysts and rediae were fixed in 96% ethanol for DNA isolation.

### DNA extraction and amplification

DNA from ethanol-fixed specimens of putative invertebrate hosts was extracted by isopropanol precipitation as described [1]. DNA from individually fixed trematode intermediate stages was isolated with the NucleoSpin Tissue XS kit (Macherey-Nagel, Düren, Germany) according to the manufacturer’s instructions.

Two nuclear ribosomal DNA loci were amplified. First, we designed a PCR test named CF700 with the primers (Table 2) directed to the regions of ribosomal DNA under the following conditions: 1) the 3′ nucleotide of the primer consisted of a base that did not pair with any ribosomal DNA sequence publicly available in the GenBank database on 26-Apr-2012, and 2) the primer sequence differed by at least 11 and 5 nucleotide substitutions, respectively, from any ribosomal DNA sequence publicly available in the GenBank database on 26-Apr-2012. As a template, we used the *C. faba* sequence of 18S rDNA available under NCBI acc. No. JQ231122. NCBI Blast revealed *Euryhelmis costaricensis* AB521797 and other Opisthorchiidae species as the most similar sequence available at the date of the PCR test design (note that more trematode DNA sequences were available in the GenBank database at the time of manuscript submission, particularly those of the phylogenetically more closely related family Pleurogenidae). The expected product size was 372 bp, corresponding to nucleotides 756–1127 of JQ231122. Products of a different length were not considered positive outcomes of the proposed CF700 test.

**Table 2:** **PCR primers used**

**Locus**	**Primer name**	**Primer sequence**
CF700 (18S rDNA)	CF700fw	TGA CGG TTC TAC CGT TAG CAT GCT TCC G
	CF700rv	GGG GAC TGC CCG TGG GGT CAC TTG
ITS2 and flanking 5.8S and 28S rDNA	NC13	ATC GAT GAA GAA CGC AGC
	Dd28SR1	ACA AAC AAC CCG ACT CCA AG

Because we found the selected 18S rDNA locus to contain too low variability to distinguish between the closely related species, we next amplified the full-length sequence of the internal transcribed spacer 2 (ITS2) and the flanking 5.8S and 28S rDNA regions using primers designed for *C. faba* nuclear rDNA segment 5 as described [1,24,25].

### DNA sequencing and analysis

The obtained PCR products were purified using USB Exo-SAP-IT (Affymetrix, Santa Clara, CA) and subjected to bidirectional Sanger sequencing using an ABI 3730xl DNA Analyzer (Applied Biosystems, Foster City, CA). All the obtained sequences were submitted to the GenBank database under accession numbers KC543332-KC543350 and KM594114-KM594185, and their IDs are provided in Tables 3 and 4. The sequences were analyzed using the NCBI Blast algorithm. Because we recently solved the taxonomic position of *C. faba* [1], we did not perform any detailed phylogenetic analyses except for the calculation for evolutionary divergence ratios of the newly obtained sequences.

**Table 3:** **The PCR-based diagnostic test CF700 revealed the presence of** ***C. faba*** **or closely related species in benthos obtained at sites of focal occurrence of adult** ***C. faba*** **individuals**

**Species**	**Sampling site and date**	**Total N examined**	**Positive**	**Prevalence [%]**	**Voucher IDs of CF700-positive specimens (NCBI GenBank IDs)**
Mollusca:					
*Bythinella austriaca*	1	59	0	0	-
*Bythinella austriaca*	2	10	1	10	1066 (KM594114)
Turbellaria:					
*Dendrocoelum lacteum*	1	1	0	0	-
*Dugesia gonocephala*	1	4	0	0	-
Nematoda:					
*Gordius* sp.	1	3	0	0	-
Crustacea:					
*Gammarus fossarum*	1	121	19	16	250, 252, 257 (KC543332), 260 (KC543333), 266, 267, 272, 275, 283, 287, 288, 291, 198, 305, 306, 307, 316, 318, 319 (KC543334)
*Gammarus fossarum*	2	20	0	0	-
*Gammarus fossarum*	6	81	1	1	486 (KC543343)
Plecoptera:					
*Isoperla grammatica*	1	18	2	11	776, 786 (KC543346)
*Isoperla* sp.	2	20	11	55	983-990, 991 (KC543349), 992 (KC543350), 1009
*Leuctra braueri*	1	3	0	0	-
*Leuctra prima*	1	1	0	0	-
*Leuctra teriolensis* × *rauscheri*	1	3	0	0	-
*Perla bipunctata*	6	1	0	0	-
*Perla bipunctata*	1	24	10	42	799, 800, 804, 806, 808, 818 (KC543347), 820, 822 (KC543348), 823, 826
*Perla burmeisteriana*	6	3	0	0	-
*Perla* sp.	2	20	6	30	1047, 1049, 1053, 1058, 1059, 1062
*Protonemura auberti*	6	7	0	0	-
Ephemeroptera:					
*Baetis alpinus*	1	17	0	0	-
*Ecdyonurus venosus*	1	26	11	42	369, 376 (KC543339), 379 (KC543340), 381, 384, 387, 389, 391 (KC543341), 392, 393, 394
*Ecdyonurus venosus*	6	18	0	0	-
*Ecdyonurus* sp., juv.	6	4	0	0	-
*Epeorus assimilis*	6	1	0	0	-
*Habroleptoides modesta*	1	1	0	0	-
*Rhithrogena carpatoalpina*	6	1	0	0	-
*Rhithrogena picteti* × *iridina*	1	83	27	33	325, 326, 327, 330, 332 (KC543335), 333 (KC543336), 335 (KC543337), 336 (KC543338), 339, 340, 342, 349, 350, 353, 356, 359, 360, 362, 363, 364, 365, 367, 430 (KC543342), 651 (KC543344), 668, 694, 695 (KC543345)
*Rhithrogena* sp.	2	20	2	10	-
*Seratella ignita*	6	1	0	0	-
Trichoptera:					
*Drusus discolor*	1	1	0	0	-
*Drusus* sp.	1	5	0	0	-
*Hydropsyche instabilis*	6	1	1	100	572
*Philopotamus ludificatus*	6	4	0	0	-
*Plectrocnemia conspersa*	6	7	0	0	-
*Potamophylax luctuosus*	6	3	0	0	-
*Potamophylax* sp., juv.	6	2	0	0	-
*Rhyacophila hirticornis*	1	1	0	0	-
*Rhyacophila polonica*	1	12	0	0	-
*Rhyacophila polonica*	6	1	0	0	-
*Rhyacophila tristis*	1	1	0	0	-
*Rhyocophila hirticornis*	1	3	0	0	-
*Wormaldia copiosa*	1	3	0	0	-
Diptera:					
*Dicranota* sp.	1	1	0	0	-
*Hexatoma* sp.	1	1	0	0	-
Coleoptera:					
*Elmis aenea*	6	1	0	0	-
Aves (controls from definitive hosts):					
*Hirundo rustica*					827 (KM594115)
*Erithacus rubecula*					865 (KM594116)
*Sylvia atricapilla*					866 (KM594117)
*Regulus regulus*					870 (KM594118)

Sampling sites and dates are indicated according to Table 1. We denoted any tests which yielded a PCR product of the expected length (~402 bp) as positive. Lack of PCR product or PCR products of different length were considered negative tests. Representative PCR products were subjected to Sanger sequencing. The NCBI GenBank accession numbers are indicated (KC543332-KC543350, KM594114-KM594118). The single-band PCR products of unexpected size were sequenced as well, but none of them correspond to any sequence available in the NCBI GenBank database.

**Table 4:** **Genetic identification of** ***C. faba*** **among the representative pool of trematode specimens isolated from benthic invertebrates in the Veľká Fatra Mts.**

**Group, species and NCBI accession No. with the highest similarity**						
**Specimen ID**	**Morphospecies according to** [5,23]	**Sampling site and date according to Table** 1	**Host**	**Positivity in the CF700 test**	**GenBank accession no. of ITS2 and flanking rDNA regions**	**Estimated evolutionary divergence from the species with the highest sequence similarity**
***Collyriclum faba*** **(Collyriclidae); similar to the** ***C. faba*** **isolate from** ***Saxicola rubetra*** **JQ231122**						
1467	Lecithodendriidae	5	*Bythinella austriaca*	1	KM594156	0.009
1473	Immature cercaria	5	*Bythinella austriaca*	1	KM594161	0.009
1552	Lecithodendriidae	5	*Bythinella austriaca*	1	KM594176	0.009
1556	Lecithodendriidae	5	*Bythinella austriaca*	1	KM594180	0.009
1558	Lecithodendriidae	5	*Bythinella austriaca*	1	KM594182	0.009
391	N/A (lysate of the host)	1	*Ecdyonurus venosus*	1	KM594183	0.009
695	N/A (lysate of the host)	1	*Rhitrogena picteti × iridina*	1	KM594185	0.009
**Heterophyidae; similar to** ***Euryhelmis costaricensis*** **AB521800** ^*^						
1340	N/D	5	*Bythinella austriaca*	0	KM594133	N/D
1497	Opisthorchiidae	4	*Bythinella austriaca*	1	KM594168	0.024
1553	Opisthorchiidae	4	*Bythinella austriaca*	0	KM594177	0.024
**susp. Microphallidae; similar to** ***Maritrema madrynense*** **KF575167**						
1471	Microphallidae	5	*Bythinella austriaca*	1	KM594159	0.106
1498	Microphallidae	5	*Bythinella austriaca*	1	KM594169	0.106
1549	Microphallidae	5	*Bythinella austriaca*	1	KM594173	0.106
1550	Immature cercaria	5	*Bythinella austriaca*	1	KM594174	0.106
**susp. Nanophyetidae / Paragonimidae; similar to** ***Paragonimus kellicotti*** **HQ900670** ^**^						
1343	N/D	5	*Bythinella austriaca*	0	KM594136	N/D
1358	N/D	3	*Perla marginata*	0	KM594150	N/D
1475	Nanophyetidae	4	*Bythinella austriaca*	1	KM594163	0.082
1477	Nanophyetidae	5	*Bythinella austriaca*	1	KM594165	0.080
**susp. Troglotrematidae; similar to Troglotrematidae sp. H2009 AB521803**						
1470	Troglotrematidae	5	*Bythinella austriaca*	1	KM594158	0.081
1496	Troglotrematidae	5	*Bythinella austriaca*	1	KM594167	0.081
1551	N/D	5	*Bythinella austriaca*	1	KM594175	0.081
1555	Troglotrematidae	4	*Bythinella austriaca*	1	KM594179	0.081
**susp. Lecithodendriidae, species 1; similar to** ***Collyriclum faba*** **JQ231122**						
1472	Lecithodendriidae	5	*Bythinella austriaca*	1	KM594160	0.150
1557	Immature cercaria	4	*Bythinella austriaca*	0	KM594181	0.158
**susp. Lecithodendriidae, species 2; similar to** ***Collyriclum faba*** **JQ231122**						
1468	Immature cercaria	5	*Bythinella austriaca*	1	KM594157	0.083
**susp. Lecithodendriidae, species 3; similar to** ***Collyriclum faba*** **JQ231122**						
1476	Lecithodendriidae	5	*Bythinella austriaca*	1	KM594164	0.060
1495	Lecithodendriidae	4	*Bythinella austriaca*	1	KM594166	N/D
1547	Immature cercaria	5	*Bythinella austriaca*	1	KM594171	0.060
1548	Immature cercaria	4	*Bythinella austriaca*	1	KM594172	0.060
**susp. Lecithodendriidae, species 4; similar to** ***Collyriclum faba*** **JQ231122**						
1554	Immature cercaria	5	*Bythinella austriaca*	1	KM594178	0.068
**susp. Lecithodendriidae, species 5; similar to** ***Collyriclum faba*** **JQ231122**						
486	N/A (lysate of the host)	6	*Gammarus fossarum*	0	KM594184	N/D
**susp. Lecithodendriidae, species 6; similar to** ***Collyriclum faba*** **JQ231122**						
1466	Immature cercaria	5	*Bythinella austriaca*	1	KM594155	0.064
1474	Lecithodendriidae	5	*Bythinella austriaca*	1	KM594162	0.064
1499	Lecithodendriidae	4	*Bythinella austriaca*	1	KM594170	0.064
**susp. Lecithodendriidae, species 7; similar to** ***Collyriclum faba*** **JQ231122**						
1324	N/D	3	*Perla marginata*	1	KM594119	0.073
1325	N/D	3	*Perla marginata*	1	KM594120	0.073
1326	N/D	3	*Perla marginata*	1	KM594121	0.073
1327	N/D	3	*Isoperla* sp.	1	KM594122	0.073
1328	N/D	3	*Perla marginata*	1	KM594123	0.073
1329	N/D	3	*Perla marginata*	1	KM594124	0.073
1331	N/D	3	*Perla marginata*	1	KM594125	0.073
1332	N/D	3	*Perla marginata*	1	KM594126	0.073
1333	N/D	3	*Perla marginata*	1	KM594127	0.073
1334	N/D	3	*Perla marginata*	1	KM594128	0.073
1335	N/D	3	*Perla marginata*	1	KM594129	0.073
1337	N/D	3	*Perla marginata*	1	KM594130	0.073
1338	N/D	3	*Perla marginata*	1	KM594131	0.073
1339	N/D	3	*Perla marginata*	1	KM594132	0.073
1341	N/D	3	*Perla marginata*	1	KM594134	0.073
1342	N/D	3	*Perla marginata*	1	KM594135	0.073
1344	N/D	3	*Perla marginata*	1	KM594137	0.073
1345	N/D	3	*Perla marginata*	1	KM594138	0.073
1346	N/D	3	*Perla marginata*	1	KM594139	0.073
1347	N/D	3	*Perla marginata*	1	KM594140	0.073
1348	N/D	3	*Perla marginata*	1	KM594141	0.073
1349	N/D	3	*Perla marginata*	1	KM594142	0.073
1350	N/D	3	*Perla marginata*	1	KM594143	0.073
1351	N/D	3	*Perla marginata*	1	KM594144	0.073
1352	N/D	3	*Perla marginata*	1	KM594145	0.073
1353	N/D	3	*Perla marginata*	1	KM594146	0.073
1354	N/D	3	*Perla marginata*	1	KM594147	0.073
1356	N/D	3	*Perla marginata*	1	KM594148	0.073
1357	N/D	3	*Perla marginata*	1	KM594149	0.073
1359	N/D	3	*Perla marginata*	1	KM594151	0.073
1363	N/D	3	*Perla marginata*	1	KM594152	0.073
1364	N/D	3	*Perla marginata*	1	KM594153	0.073
1365	N/D	3	*Perla marginata*	1	KM594154	0.073

*NCBI Blast revealed 98% sequence similarity to *Euryhelmis costaricensis* AB521800, but only ≤91% sequence similarity to any of the Opisthorchiidae species. **There were no publicly available ITS2 sequences of Nanophyetidae at the time when the NCBI Blast search was performed (2-Apr-2014).

The internal transcribed spacer 2 (ITS2) and the flanking 5.8S and 28S rDNA regions were sequenced. The NCBI GenBank accession numbers are indicated (KM594119-KM594185).

GenBank BLASTn was performed with the following parameters: expected number of chance matches in a random model = 10, length of seed that initiates an alignment = 11, reward for matching bases = 1, penalty for mismatching bases = −3, gap cost: existence = 5, extension = 2. The search was limited to the Trematoda only. The newly obtained sequences and those with the highest similarity as revealed by the NCBI Blast algorithm were imported in MEGA5. The sequences were trimmed to ensure 100% coverage of the sequences analyzed in each batch, including the sequences of outgroups, and were aligned by the ClustalW algorithm. The *C. faba* and *C. faba*-like sequences from *Perla marginata*, Heterophyidae, Microphallidae, Nanophyetidae and Paragonimidae, and Troglotrematidae were trimmed and analyzed separately.

The alignments were manually checked for any inconsistencies. Maximum likelihood fits of 24 nucleotide substitution models were performed. These included General Time Reversible, Hasegawa-Kishino-Yano, Tamura-Nei, Tamura 3-parameter, Kimura 2-parameter, and the Jukes-Cantor model, each alone or in combination with modeling of the non-uniformity of evolutionary rates among sites using discrete Gamma distribution with five rate categories and/or by assuming that a certain fraction of sites are evolutionarily invariable [26]. For each model, Bayesian information criterion, Akaike information criterion (corrected), and maximum likelihood values were calculated (Additional file 1: Tables S1-S6).

The best fit model was identified based on the Bayesian information criterion according to the evidence-based suggestion by Tamura et al. [27] and with a low number of parameters to keep the variance as low as possible. Following the determination of the best fit model, the mean evolutionary divergence of the sequences was estimated. The analyses were conducted using the maximum composite likelihood model, where the differences in the composition bias among sequences were considered in evolutionary comparisons. We calculated the number of base differences per site by averaging over all sequence pairs between groups (distance) ± SE using the bootstrap procedure at 10,000 replicates. The included codon positions were 1st + 2nd + 3rd + noncoding. All positions containing gaps and missing data were eliminated.

For the sequences of *C. faba*, the Kimura 2-parameter model [28] was used with a gamma distribution (shape parameter = 1) to model the rate of variation among sites. The analysis involved 17 nucleotide sequences, a total of 579 positions in the final dataset. The sequences of adult *C. faba* (JQ231122) from *Saxicola rubetra* and *Paramacroderoides kinsellai* (HM137665) were used as outgroups.

For the *C. faba*-like sequences, the Tamura-Nei model [29] was used with a gamma distribution (shape parameter = 1) to model the rate of variation among sites. The analysis involved 49 nucleotide sequences, a total of 565 positions in the final dataset. The sequences of adult *C. faba* (JQ231122) from *Saxicola rubetra* and *Paramacroderoides kinsellai* (HM137665) were used as outgroups.

For the sequences of Heterophyidae, the Kimura 2-parameter model [28] was used. The analysis involved 4 nucleotide sequences, a total of 458 positions in the final dataset. The sequences of *Clonorchis sinensis* (KJ137227) and *Euryhelmis costaricensis* (AB521800) were used as outgroups.

For the sequences of Microphallidae, the Kimura 2-parameter model [28] was used. The analysis involved 5 nucleotide sequences, a total of 529 positions in the final dataset. The sequence of *Maritrema madrynense* (KF575167) was used as the outgroup.

For the sequences of Nanophyetidae and Paragonimidae, the Kimura 2-parameter model [28] was used. The analysis involved 3 nucleotide sequences, a total of 413 positions in the final dataset. The sequence of *Paragonimus kellicotti* (HQ900670) was used as the outgroup.

For the sequences of Troglotrematidae sequences, the Kimura 2-parameter model [28] was used. The analysis involved 5 nucleotide sequences, a total of 652 positions in the final dataset. The sequence of Troglotrematidae sp. HS-2009 (AB521803) was used as the outgroup.

### Statistical analyses of infection rates

The expected weighted rate of simultaneous infection by two trematode species “wr_2sp_” was calculated as follows:

$$ w{r}_{2sp}=\frac{{\displaystyle {\sum}_{i=6}}\left(\frac{N_{inf}-{N}_{inf\_by\_ spec\_i\ }}{N_{hosts\ }} \times {N}_{inf\_by\_ spec\_i\ }\right)}{N_{inf}}\times 100 $$

“i” = number of operational taxonomic units (such as morphologically identified trematode families); “N_inf_by_spec_i_" = the number of hosts infected with one or more individuals of a particular trematode species; “N_inf_” = N_inf by spec i_ summarized for all the trematode species found; “N_hosts_” = total number of host individuals examined.

## Results

### Identification based on amplification of conserved rDNA region

The CF700 PCR-based diagnostics of genomic DNA isolated from benthic organisms obtained at sites of occurrence of adult *C. faba* revealed high prevalence of *C. faba* or closely related organisms in a wide range of benthic species. The first intermediate host of *C. faba* was expected to be mollusks [4]. The examined streams hosted a species-poor assemblage of mollusks (Mollusca), which were represented by a single but abundant species, *Bythinella austriaca*. The CF700 test was positive only in one of 69 *B. austriaca* individuals, suggesting a rather low prevalence of *C. faba* in this species (Table 3).

The second intermediate host of *C. faba* was expected to be an insect [4]. We thus focused on the benthic arthropods of the examined streams. A single but highly abundant crustacean species (Crustacea), *Gammarus fossarum*, was identified. Twenty of the 221 *G. fossarum* individuals examined were positive in the CF700 test. We also collected at least nine species of stoneflies (Plecoptera), most of which were identified to species level. *Isoperla grammatica*, *Isoperla* sp., *Perla bipunctata* and *Perla* sp. were positive in the CF700 test at up to 55% prevalence (Table 3). Further, we collected at least eight species of mayflies (Ephemeroptera), most of which were identified to species level. Of them, *Ecdyonurus venosus* and *Rhithrogena picteti x iridina* were positive in the CF700 test at up to 42% prevalence, whereas *Baetis alpinus* was negative in the CF700 test despite the relatively high number of individuals tested (n = 17). We also collected at least ten species of caddisflies (Trichoptera), most of which were determined to the species level. Only the single examined individual of *Hydropsyche instabilis* was positive in the CF700 test. However, the total number of individuals of Trichoptera examined was relatively low, and, except for *Rhyacophila polonica*, few specimens were examined for each species. Finally, we examined two specimens of Diptera and a single specimen of Coleoptera, all of which were negative in the CF700 test (Table 3).

Selected specimens that were positive in the CF700 test (representing each host species except *Hydropsyche instabilis*) were subjected to DNA sequencing to corroborate the diagnosis of *C. faba*. In addition, the CF700 locus was sequenced in adults of *C. faba* of all the three major ecotypes, represented by cysts located in the abdominal area (*Hirundo rustica*, *Sylvia atricapilla*), at the femur (*Erithacus rubecula*) and near the coccygeal gland (*Regulus regulus*). Sequencing of PCR products of mutually identical sizes resulted in sequences similar to each other with the exception of a C/T polymorphism at the site corresponding to nt. 72 of KC543332. Importantly, whereas the majority of the newly obtained sequences contained the C allele, specimens of the adult *C. faba* of all three major ecotypes coded for the T allele (data not shown). Because we expected nil heterogeneity or heterogeneity indicating differences similar to those present within the sequences of the three ecotypes tested, we newly hypothesized that the CF700 PCR-based test might also amplify closely related species, with sequences not included in the GenBank database, making the newly designed PCR method not selective over them.

### Identification of the first intermediate host, *Bythinella austriaca*

#### November sampling

We analyzed 601 ethanol-fixed *B. austriaca* individuals obtained on 16-Nov-2013 at two sites located within the same stream separated by 700 m and differing in altitude by 70 m a.s.l. The specimens were lysed and subjected to DNA isolation and subsequent CF700 PCR-based diagnostics. Amplification of 23 specimens (3.8%) resulted in a PCR product of the expected size. The prevalence of CF700-positive specimens was similar at both sampling sites (15/387 and 10/214).

We next dissected 1320 freshly collected *B. austriaca* individuals obtained in parallel on 16-Nov-2013 at the same two sampling sites described above. The total prevalence of morphologically identified trematodes differed strongly between these sites. Only 24 (4.2%) of the 576 *B. austriaca* individuals examined were positive for trematodes at the lower sampling site. In contrast, 126 (16.9%) of 744 *B. austriaca* individuals from the upper sampling site were positive for trematodes (OR 0.21, 95% CI 0.13 – 0.33, z = 6.71, *p* < 0.01). However, the spectrum of trematode families identified based on their morphology was similar, so the individuals obtained from these two sites were analyzed together. The majority of specimens found shared the morphological characters of the family Lecithodendriidae (98 specimens, 65%). Several specimens belonged to the families Troglotrematidae (9 specimens, 6%), Nanophyetidae (7 specimens, 5%), Opisthorchiidae (5 specimens, 3%) and Microphallidae (4 specimens, 3%). The remaining 28 specimens (19%) consisted of immature cercariae, rediae or sporocysts, which could not be identified. We found only one double infection (by Opisthorchiidae gen. sp. and Lecithodendriidae gen. sp. 2); in all other cases, the infected snail hosted only one trematode species as identified according to [5]. Because the mean infection rate was 11.4%, and the expected weighted rate of simultaneous infection by two trematode species reached 6.2%, the frequency of multiple infections in *B. austriaca* was significantly lower than expected from the weighted rate of simultaneous infection by two trematode species (OR 0.11, 95% CI 0.01-0.84, z = 2.1, *p* = 0.03).

Representative specimens of each of the above families were then subjected to the CF700 PCR-based diagnostics to compare its outcomes with the morphological identification (Table 4). With the exception of one of the two specimens of Opisthorchiidae tested and single unknown immature cercariae, all trematode specimens (27 of 29) were positive in the CF700 test. This finding urged the use of a less conserved locus for the genetic characterization of the specimens. Thus, we chose a hypervariable ITS2 locus to sequence all isolated trematode specimens initially tested with the CF700 test to match the sequences obtained with those available in the NCBI GenBank database.

The full-length ITS2 and the flanking conserved 5.8S and 28S rDNA region sequences suggested that all specimens identified based on their morphology as Lecithodendriidae according to [5] share the highest similarity with *Collyriclum faba* JQ231122. Five specimens (#1467, #1473, #1552, #1556, #1558) displayed nil sequence heterogeneity and differed only by 0.009 base substitutions per site from *C. faba* JQ231122. However, they differed more from the ITS2 sequences of the other ecotypes of *C. faba*, and they differed by 0.141 base substitutions per site from the species with second highest DNA similarity, *Paramacroderoides kinselai* HM137665 from the Macroderoidideae family. Thus, we propose that these five specimens represent the cercariae (all five specimens) and sporocysts (#1558) of *C. faba. Bythinella austriaca* is thus confirmed as the first intermediate host of *Collyriclum faba*.

In addition to the five specimens displaying very close similarity to *C. faba* JQ231122, there were numerous specimens (#1466, #1468, #1472, #1474, #1476, #1499, #1547, #1548, #1552 and #1557) also identified based on their morphology as Lecithodendriidae according to [5] (or as immature cercariae of unknown origin) and that were most similar to *C. faba* JQ231122 over the other publicly available ITS2 sequences. However, they displayed high sequence variability, with the individual evolutionary divergence reaching 0.000 – 0.163 base substitutions per site. Overall, their evolutionary divergence from *C. faba* JQ231122 reached 0.060 – 0.158 base substitutions per site. Their evolutionary divergence from the second nearest species based on the sequences of ITS2 and flanking regions, *Paramacroderoides kinselai* HM137665, reached 0.134 – 0.188 base substitutions per site. For these specimens, we cannot exclude the possibility that they represent a closely related but different species without an ITS2 sequence in the GenBank database.

The obtained sequences confirmed the morphological identification of specimens from the family Microphallidae (specimens #1471, #1498, #1549 and #1550), which displayed the highest sequence similarity to *Maritrema madrynense* KF575167. However, the relatively large distance between the sequenced specimens (with nil sequence heterogeneity) and *M. madrynense* KF575167 (number of base substitutions per site 0.106) suggests that they represent a different species with sequences not yet available in the GenBank database.

Similarly, the obtained sequences confirmed the morphological determination of specimens from the family Troglotrematidae (#1470, #1496, #1551, #1555), which displayed the highest sequence similarity to Troglotrematidae sp. H2009 AB521803. However, the relatively high distance between the sequenced specimens (with nil sequence heterogeneity) and Troglotrematidae sp. H2009 AB521803 (number of base substitutions per site 0.081) suggests that they represent a different species with sequences not yet available in the GenBank database.

The specimens identified as Opisthorchiidae gen. sp. according to Faltýnková and Literák [5] (#1497, #1553) displayed the highest sequence similarity to *Euryhelmis costaricensis* AB521800 of the closely related family Heterophyidae. The two specimens sequenced differed by 0.002 base substitutions per site. Both of them differed by 0.024 base substitutions per site from the sequence of *E. costaricensis* AB521800. Among the ITS2 DNA sequences of the family Opisthorchiidae available in the GenBank database, the two specimens displayed the highest similarity to *Clonorchis sinensis* KJ137227. However, the estimated evolutionary divergence was nearly four times larger than those from the heterophyid specimens, and reached 0.081 and 0.083 base substitutions per site. Based on the combined morphological and genetic assessment, these specimens represent *Euryhelmis* sp. They differ from the common European species *E. squamula* in that they have a broader body, no eye-spots and the presence of three (instead of two) rows of large acicular spines near the dorsal lip of the mouth opening, which are consistent with *E. zelleri* described by Grabda-Kazubska [30] from a Polish mountain range located just across the border.

We were unable to confirm the identification of the specimens of the family Nanophyetidae (#1475, #1477) because there was no ITS2 sequence of any nanophyetid species available in the GenBank database (as of 18-Sep-2014). Thus, in line with the taxonomic position of Nanophyetidae, the nanophyetid specimens displayed the highest sequence similarity to the family Paragonimidae, namely to *Paragonimus kellicotti* HQ900670. The specimens displayed intraspecific heterogeneity of 0.005 base substitutions per site, whereas the estimated evolutionary divergence from *P. kellicotti* HQ900670 was 0.080 and 0.082 base substitutions per site, respectively.

#### June sampling

On 20-Jun-2013, we collected another 339 specimens of *B. austriaca*, and allowed the cercariae to emerge at 4°C for a period of 14 days. Rediae (#1340) were harboured by only a single snail, and were identified based on their morphology and ITS2 sequence as *Euryhelmis* sp., similar to the above-mentioned specimens #1497 and #1553 (Table 4). After two weeks, 328 of the initial 339 snails were dissected. Rediae and cercariae (#1343) were only found in a single snail, and were identified based on their morphology and ITS2 sequence as members of the family Nanophyetidae, similarly to the above-mentioned specimens #1475 and #1477.

The total prevalence of trematodes in *B. austriaca* collected in June was 0.3%, significantly lower than the prevalence in November (OR 0.05, 95% CI 0.01 – 0.21, z = 4.1, *p* < 0.01). In June, we could not find any trematodes hosted by *B. austriaca* that resembled *C. faba* in morphology or ITS2 sequence.

### Identification of the second intermediate hosts, *Ecdyonurus venosus* and *Rhithrogena picteti × iridina*

#### Morphological examination of the second intermediate hosts from ethanol-fixed material

Randomly selected ethanol-fixed specimens collected on 3-Aug-2012 that were not processed and lysed as described in chapter 3.1 were examined morphologically for the presence of trematode developmental stages (metacercariae). The examined material included *Gammarus fossarum*, immature stages of *Rhitrogena* spp., *Isoperla* spp. and *Perla marginata*, and imagos of *Perla marginata*, all collected on 3-Aug-2012 at two nearby sampling sites (Table 5). Morphologically, we were able to distinguish between four types of metacercariae, three morphotypes of which we found in the walls of the abdomen of larval and adult *P. marginata*. Additionally, we found trematodes of a single morphotype in the abdomen and thorax of two Plecoptera gen. sp. immature individuals (Table 5). The total prevalence of trematode intermediate stages was 50% in immature *P. marginata* hosts, 47% in imagos of the same species, 6% in *Isoperla* spp., and 0% in *Rhitrogena* spp. and *G. fossarum*. Co-infections of *P. marginata* by multiple morphotypes were frequently observed (null hypothesis rejected at OR 91.5, 95% CI 4.8 – 1754.6, z = 3.00, *p* < 0.01; random distribution hypothesis was not rejected at OR 1.7, 95% CI 0.5 – 6.4, z = 0.79, *p* = 0.43, n = 20 independent infection events in 27 host individuals).

**Table 5:** **Morphological identification of trematode intermediate stages in ethanol-fixed specimens from cohorts examined in part by DNA sequencing in Table** 3 **, collected at sampling site 2 (as defined in Table** 1 **)**

**Species**	**N examined**	**N positive**	**Prevalence [%]**	**Specification of metacercariae**	**N of individuals**	**Number of metacercariae per host individual**	**Location in the host**
*Gammarus fossarum*	167	0	0				
*Rhitrogena* spp. (immature)	101	0	0				
*Isoperla* spp. (immature)	35	2	6	Large	1	2	Abdomen
				Large	1	1	Thorax
*Perla marginata* (immature)	12	6	50	Very large	6	1.3 ± 0.5	Abdomen
				Medium	1^*^	1	Abdomen
*Perla marginata* (imago)	15	7	47	Very large	6	3.0 ± 1.1	Abdomen
				Medium	1^**^	1	Abdomen
				Small	6^†^	4.8 ± 3.1	Abdomen

^*^Mixed infection of the larva of *Perla marginata* with a single medium-sized metacercaria and two very large metacercariae. ^**^Mixed infection of the imago of *Perla marginata* with a single medium-sized, four very large and nine small metacercariae. † Mixed infection of imagos of *Perla marginata* with small and very large metacercariae in five of the six cases.

The dimensions of the four types of metacercariae found were as follows: **Very large metacercariae** (host: *Perla marginata*, n = 24): cyst 309 ± 21 × 308 ± 18 μm (range 271–342 μm), body 200 ± 19 × 200 ± 20 (range 165–253 μm), cyst wall thickness 41.7 ± 8.9 μm (range 22–65 μm). In two cases, we identified the outer and inner cyst wall, measuring 19–37 and 15–20 μm, respectively. **Small metacercariae** (host: *Perla marginata*, n = 13): cyst 119 ± 11 × 121 ± 11 μm (range 97–142 μm), body 96 ± 9 × 96 ± 8 (range 80–114 μm), cyst wall thickness 11.6 ± 2.8 μm (range 7–17 μm). In one case, we identified the outer and inner cyst wall, measuring 8–9 and 5–7 μm, respectively. **Medium-sized metacercariae** (host: *Perla marginata*, n = 2): cyst 274 × 273 μm and 180 × 174 μm, body 205 × 193 μm and 129 × 115 μm, cyst wall thickness 26–30 μm and 14–18 μm. **Large metacercariae** (host: *Isoperla* sp., n = 2): cyst 150 × 145 μm and 155 × 151 μm, body 121 × 114 μm and 127 × 124 μm, cyst wall thickness 11–12 μm and 8–9 μm. The dimensions of metacercariae recorded in immature and mature *Perla marginata* did not differ from each other and were thus analyzed as pooled cohorts.

#### Genetic examination of ethanol-fixed specimens of the second intermediate hosts

Representative CF700-positive DNA specimens from *Gammarus fossarum*, *Ecdyonurus venosus* and *Rhithrogena picteti × iridina*, obtained in June 2011 as described in chapter 3.1, were subjected to ITS2 locus sequencing. Sequencing of specimens #391 (host: *Ecdyonurus venosus*; KM594183) and #695 (host: *Rhithrogena picteti × iridina*; KM594185) revealed the ITS2 locus 99% identical with the ITS2 locus of adult specimen of *Collyriclum faba* previously isolated from *Saxicola rubetra* (JQ231122). The sequences obtained were identical to those from *B. austriaca* specimens #1467, #1473, #1552, #1556 and #1558. *Ecdyonurus venosus* and *Rhithrogena picteti × iridina* were thus confirmed as the second intermediate hosts of *C. faba*.

Sequencing of the specimen #486 (host: *Gammarus fossarum*) revealed as well an ITS2 sequence (KM594184) with the highest similarity to *C. faba* compared to other trematode species. However, it was only 94% similar, and may thus represent another trematode species with an as yet unknown ITS2 DNA sequence.

#### Genetic diversity within the CF700-positive isolates (June sampling)

On 20-Jun-2013, we collected and immediately dissected 234 arthropods, focusing predominantly on species that were positive for *C. faba* or *C. faba*-like trematodes in the previously performed CF700 tests (Table 3). The obtained metacercariae were photographed, identified based on their morphology, and tested in the CF700 test. The ITS2 locus was amplified and sequenced from individuals positive in the CF700 test.

We examined 101 larvae and 6 adults of *Perla marginata*. Fifty-six larvae (55%) and two imagos (33%) were positive for at least one metacercaria. The mean intensity of infection was 2.0 ± 1.3 individuals per host (range 1–5). Most frequently, the trematodes were located in the abdomen (49 cases, including both infected imagos), in five cases, the trematodes were in the abdomen and thorax; in three cases, the trematodes were found in the thorax only; and in one case, the trematodes were found in the head. We sequenced the ITS2 locus of 34 trematodes, representing all locations within the hosts’ body and all size categories found, 33 of which were found in the *P. marginata* larvae and one of which originated from the adult. The single specimen from adult *P. marginata* (#1358) was identified as Nanophyetidae gen. sp. based on its ITS2 sequence similarity to the above-described specimens #1343, #1475 and #1477. The ITS2 sequences of all 33 specimens isolated from the larvae displayed nil evolutionary distance. The ITS2 sequence of these 33 specimens was unique and was not found in any of the sequenced isolates from *B. austriaca*. It did not match those of *C. faba* JQ231122 (evolutionary distance 0.073 base substitutions per site), other specimens of *C. faba* identified in *B. austriaca* (0.071 base substitutions per site), or *C. faba*-like specimens isolated from *B. austriaca* (0.011 – 0.175 base substitutions per site). The ITS2 locus of these 33 specimens differed by 0.151 base substitutions per site from the ITS2 locus of the species with second highest DNA similarity, *Paramacroderoides kinselai* HM137665.

We examined 15 larvae and 39 adults of *Isoperla* spp. Only one of the larvae examined and none of the adults were positive for any trematodes. The single trematode obtained from the abdomen was negative in the CF700 test and thus was not subjected to ITS2 amplification and sequencing.

We also examined 46 larvae of Ephemeroptera gen. sp., one larva of Trichoptera, four individuals of *Gammarus fossarum*, 18 other aquatic insects and 5 adult turbellarian specimens, all of which were negative for trematodes.

### Description of cercariae of *C. faba* (Figure 1)

**Figure 1:**
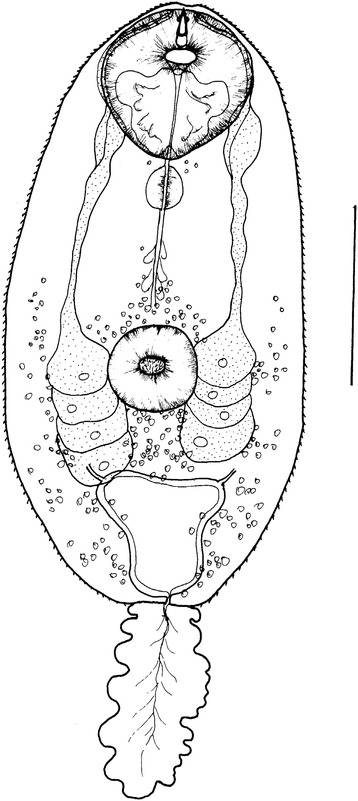
**Cercaria of** ***Collyriclum faba*** **(Bremser in Schmalz, 1831).** Scale bar 50 μm.

Synonymum: Lecithodendriidae gen. sp. 2 of [5].

First intermediate host: *Bythinella austriaca* (Frauenfeld, 1857)

Site: hepatopancreas

Locality: Veterné, Veľká Fatra, Slovakia

Small virgulate xiphidiocercariae. Body oval, with maximum width at level of ventral sucker, length 90–135 (104), width 34–59 (39). Whole body covered with minute spines, becoming smaller posteriorly. Oral sucker 23–39 (28) in diameter, ventro sub-terminal, rounded, larger than ventral sucker. In a posterior half of oral sucker a large, lobed triangular virgula. Rather stout stylet dorsal to mouth opening; with sharp tip and prominent anterior thickening; not sclerotized in posterior part, 13–15 (14) long. Ventral sucker 14–27 (17) in diameter, spherical, postequatorial, central pit armed with small spines. Prepharynx short; pharynx oval, weakly muscular. Esophageal primordium long, inconspicuous, surrounded by small cells of unknown origin; intestinal bifurcation and caeca not observed. Four pairs of penetration gland-cells reaching from mid-level of ventral sucker below its posterior border; transparent, with well-visible cell nuclei; each gland with own lateral outlet, opening near tip of stylet. Excretory vesicle trapeze-shaped, thick-walled, main collecting channels opening in antero-lateral corners. Six pairs of flame cells. Refractive granula scattered in body, more numerous in posterior half. Tail highly contractile, shorter than body, 41–85 (64) long, 9–18 (12) wide.

Remarks: The present virgulate xiphidiocercariae fit well within the ‘lecithodendriid-like’ group of trematodes (Pleurogenidae, Lecithodendriidae, Gyrabascidae, Phaneropsolidae and Leyogonimidae) [31] in the presence of a characteristic virgula in the posterior half of oral sucker. The virgula is a paired residual reservoir of mucous secretions of degenerated gland-cells in body. It is considered as an important characteristic of cercariae of the above-mentioned families, which form a monophyletic clade with other families (e.g., Microphallidae, Prosthogonimidae) designated as the superfamily Microphalloidea Ward, 1901 [31]. Thus the morphology of the present cercaria corroborates the study by Heneberg and Literák [1], who found that *C. faba* segregates with the superfamily Microphalloidea.

The present cercaria is conspecific with Lecithodendriidae gen. sp. 2 of Faltýnková and Literák [5] in the shape of the stylet (relatively stout stylet with sharp tip, prominent anterior thickening, unsclerotized base) and in shape of the virgula (large, taking nearly half space in oral sucker, lobed, triangular), in the presence of four pairs of penetration gland-cells located around the ventral sucker, a trapeze-shaped excretory vesicle, and refractive granula mainly in posterior part of body. Unfortunately, no material in formalin for measurements could be obtained because of relatively few available, in part immature, cercariae were as priority used for molecular analyses. Dimensions of the cercariae features presented in this description were retrieved from [5]. Sporocysts 205–346 (244) long and 96–131 (116) wide [5].

## Discussion

Using a combined morphological and genetic approach, we elucidated the complete life cycle of *C. faba* in Central Europe*,* which is thus confirmed to include the aquatic gastropod mollusk *Bythinella austriaca* as the first intermediate host, the mayflies *Ecdyonurus venosus* and *Rhithrogena picteti x iridina* as the second intermediate hosts, and birds (primarily but not exclusively passeriform birds [1]) as the definitive hosts.

The first intermediate host, *B. austriaca*, is a least concern species according to the IUCN Red List [32]. It occurs typically in springs of tributaries of the Danube river. Its distribution is highly focal but at sites of its occurrence, it is frequently the dominant or the only aquatic snail species. It is found in the Alpine-Carpathian region (Germany, Austria, Poland, Slovakia, Hungary, Czech Republic and perhaps the Ukraine), and may thus be associated with the known endemic foci of *C. faba* in Slovakia and the Czech Republic. The helminths parasitizing *B. austriaca* were previously studied using an extensive number of specimens [5,33] but the trematodes found were never identified to the species level except for *Troglotrema acutum* and *Euryhelmis squamula*. The species complex of *Bythinella* includes several closely related species that occupy similar niches in other European mountain ranges. An ecologically and morphologically similar genus of aquatic gastropod mollusks, *Amnicola*, may serve as the first intermediate host of *C. faba* in the Eastern and North-Central United States, where endemic foci of *C. faba* are present as well.

As the second intermediate hosts, we identified the mayflies *Ecdyonurus venosus* and *Rhithrogena picteti* × *iridina*. Because these species were highly dominant among the sampled larval assemblages of Ephemeroptera (Table 3), it is possible that the list of species of Ephemeroptera serving as second intermediate hosts may be extended in the future by more thorough sampling. *Ecdyonurus venosus* (Heptageniidae; *Ephemera venosa* according to [34]) is a widely distributed Palearctic species of mountain rivers and streams especially with relatively fast flow and stony substrates. Although it tolerates varying ionic water composition, it is strongly linked to oligotrophic waters, because it is highly sensitive to acidification, eutrophication and siltation [35]. *Rhithrogena picteti* and *R. iridina* (Heptageniidae) are sibling species with limited features allowing their differentiation, particularly in their larval stages [36]. They are widely distributed across the Carpathian Mts. and the Balkans. The genus *Rhithrogena* is distributed in the Palearctic (85+ species) and Nearctic regions (22+ species). One or two species extend to the Indomalayan region [36]. Thus we hypothesize that other species of Ephemeroptera of the family Heptageniidae may serve as second intermediate hosts of *C. faba* in the Americas as well.

Although we did not collect enough data to answer fully the question of the timing of the *C. faba* life cycle, we performed sampling at multiple times throughout the year, which may shed at least some light on the intra-annual changes of the life cycle of *C. faba*. We sampled the first intermediate host, *B. austriaca*, in June and November. Of the 339 specimens examined in June, none were positive for *C. faba*. In November, we took samples from two sites at a single stream, separated by only 700 m distance and differing in altitude by 70 m a.s.l. At one of these sites, all 576 *B. austriaca* examined were negative for *C. faba*, whereas at the other site, five of 744 *B. austriaca* were positive for *C. faba* (Table 4). We sampled the putative second intermediate hosts in June and August. We confirmed the presence of *C. faba* in the specimens collected in June, but *C. faba* (and surprisingly all trematodes) were absent in the mayfly specimens collected in the August. In contrast, the specimens of Plecoptera were heavily infected by other trematode species during all sampling attempts (Tables 3, 4 and 5). It remains to be elucidated which intermediate stage survives the winter period. Because the snails of *Bythinella* are relatively long-lived, it is possible that a portion of *C. faba* may overwinter in the snails similarly to other trematode species infecting cold-living [33] as well as tropical [37] prosobranch mollusks. Among the major arguments for the slow development of sporocysts is the cold water of the mountain streams in which the development must be completed. Infections of insect larvae are expected to predominate in autumn. The trematodes survive the winter in their second intermediate host and are infectious to birds in the following spring and early summer.

In this study, we also identified numerous other trematode species hosted by the examined benthic organisms. Although most of the DNA sequencing data matched the morphological analysis of *B. austriaca*-associated trematodes performed a decade ago by A. Faltýnková and I. Literák [5], we noticed several exceptions. We confirmed the morphological identification of Troglotrematidae gen. sp. and Microphallidae gen. sp., both representing species with ITS2 DNA sequences publicly unavailable at the time of analysis. We questioned the morphological determination of the Opisthorchiidae gen. sp. because the ITS2 locus of such specimens displayed high similarity to that of *Euryhelmis costaricensis* of the closely related family Heterophyidae. We thus conclude that these specimens represent *Euryhelmis* sp., and we speculate that the available diagnostic features may be consistent with the identification of *E. zelleri* known from sampling sites just across the border with Poland [30]. The closely related species, *E. squamula*, was previously confirmed in the same host species, *B. austriaca*, in Germany [33]. For the Nanophyetidae gen. sp., we obtained sequences similar to the Paragonimidae family. Because there were no publicly available ITS2 DNA sequences at the time of analysis and because the family Paragonimidae is the most closely related family to Nanophyetidae, we assume that the morphological diagnosis of Nanophyetidae gen. sp. was correct and that our specimens represent the first sequenced ITS2 loci of the family Nanophyetidae. The Lecithodendriidae family is the most complicated. The snails of *B. austriaca* were previously suggested to harbor several morphospecies of this family, one of which turned out as *C. faba* of the family Collyriclidae. Additionally, the other sequenced members of the morphospecies identified as Lecithodendriidae gen. sp. displayed the highest similarity to *C. faba* compared to other specimens with ITS2 DNA sequences publicly available. However, because no ITS2 sequences of Lecithodendriidae species were publicly available at the time of analysis, we were unable to determine whether they indeed represent any lecithodendriid species or whether they should be re-classified.

## Conclusions

Using extensive sampling of benthic organisms, here we elucidated the life cycle of *Collyriclum faba* of the family Collyriclidae. The restricted distribution of the first intermediate host, *Bythinella austriaca*, explains the highly focal distribution of *C. faba* noticed previously by numerous authors [6,7,38] despite the wide distribution of its second intermediate and definitive host species. Utilization of both larval and adult Ephemeroptera spp. as the second intermediate hosts explains the known spectrum of definitive host species [1], with the highest prevalence in species feeding on larvae (such as *Cinclus cinclus* and *Motacilla cinerea*) or adults of Ephemeroptera (such as *Sylvia atricapilla* and *Regulus regulus*). Further research is needed to confirm the utilization of closely related species of prosobranch grastropods and mayflies at other foci of the *C. faba* distribution range worldwide and to address possible similarities with the hitherto unknown life cycle of the only other member of the family Collyriclidae, the intestinal cyst-forming helminth *Collyricloides massanae*, which some authors suggest should be synonymized with *C. faba* [12].

## Additional file

Additional file 1: Table S1. Maximum-likelihood fits of various nucleotide substitution models for the ITS2 and flanking 5.8S and 28S rDNA locus of Collyriclum faba. **Table S2.** Maximum-likelihood fits of various nucleotide substitution models for the ITS2 and flanking 5.8S and 28S rDNA locus of Collyriclum faba-like specimens. **Table S3.** Maximum-likelihood fits of various nucleotide substitution models for the ITS2 and flanking 5.8S and 28S rDNA locus of Heterophyidae. **Table S4.** Maximum-likelihood fits of various nucleotide substitution models for the ITS2 and flanking 5.8S and 28S rDNA locus of Microphallidae. **Table S5.** Maximum-likelihood fits of various nucleotide substitution models for the ITS2 and flanking 5.8S and 28S rDNA locus of Nanophyetidae and Paragonimidae. **Table 6.** Maximum-likelihood fits of various nucleotide substitution models for the ITS2 and flanking 5.8S and 28S rDNA locus of Troglotrematidae.
