# Supplementary material for: Intermediate hosts of the trematode Collyriclum faba (Plagiochiida: Collyriclidae) identified by an integrated morphological and genetic approach
Source: Parasit Vectors. 2015 Feb 8;8:85. doi: 10.1186/s13071-015-0646-3 (PMC4332736; doi:10.1186/s13071-015-0646-3)
Supplement: Additional file 1: Table S1. — Maximum-likelihood fits of various nucleotide substitution models for the ITS2 and flanking 5.8S and 28S rDNA locus of Collyriclum faba. Table S2. Maximum-likelihood fits of various nucleotide substitution models for the ITS2 and flanking 5.8S and 28S rDNA locus of Collyriclum faba-like specimens. Table S3. Maximum-likelihood fits of various nucleotide substitution models for the ITS2 and flanking 5.8S and 28S rDNA locus of Heterophyidae. Table S4. Maximum-likelihood fits of various nucleotide substitution models for the ITS2 and flanking 5.8S and 28S rDNA locus of Microphallidae. Table S5. Maximum-likelihood fits of various nucleotide substitution models for the ITS2 and flanking 5.8S and 28S rDNA locus of Nanophyetidae and Paragonimidae. Table 6. Maximum-likelihood fits of various nucleotide substitution models for the ITS2 and flanking 5.8S and 28S rDNA locus of Troglotrematidae. [file 13071_2015_646_MOESM1_ESM.pdf]

SUPPLEMENTARY MATERIALS (Parasites & Vectors)

Title: **Intermediate hosts of the trematode *Collyriclum faba* (Plagiochiida: Collyriclidae) revealed by integrative morphological and genetic approach**

Authors: **Petr Heneberg[1, *], Anna Faltýnková[2], Jiří Bizos[1], Milena Malá[1], Juraj Žiak[3], Ivan Literák[4]**

Affiliations:

[1] Third Faculty of Medicine, Charles University in Prague, Ruská 87, CZ-100 00 Prague, Czech Republic

[2] Institute of Parasitology, Biology Centre ASCR, Branišovská 31, CZ-370 05 České Budějovice, Czech Republic

[3] Administration of the Velká Fatra National Park, P. O. Hviezdoslavova 38 SK-036 01 Martin, Slovakia

[4] Department of Biology and Wildlife Diseases, Faculty of Veterinary Hygiene and Ecology, University of Veterinary and Pharmaceutical Sciences Brno, Palackého 1-3, CZ-612 42 Brno, Czech Republic

[*] Corresponding author, E-mail: petr.heneberg@lf3.cuni.cz, Tel. 00420-775311177, Fax 00420-267162658

Suppl. Table 1. Maximum-likelihood fits of various nucleotide substitution models for the ITS2 and flanking 5.8S and 28S rDNA locus of *Collyriclum faba*. The sequences were trimmed relative to the shortest sequences of the alignment. The analysis involved 17 nucleotide sequences, a total of 579 positions in the final dataset. The sequences of adult *C. faba* from *Saxicola rubetra* (JQ231122) and *Paramacroderoides kinsellai* (HM137665) were used as outgroups. Models with the lowest Bayesian information criterion (BIC) scores are considered to describe the substitution pattern the best. For each model, the corrected Akaike information criterion (AICc) value, maximum likelihood value (ln*L*), and the number of parameters (including branch lengths) are also presented. Non-uniformity of evolutionary rates among sites may be modelled using a discrete Gamma distribution (+G) with five rate categories and by assuming that a certain fraction of sites are evolutionarily invariable (+I). Whenever applicable, estimates of gamma shape parameter and/or the estimated fraction of invariant sites are shown. Assumed or estimated values of transition/transversion bias (*R*) are also shown for each model. They are followed by nucleotide frequencies and rates of base substitutions (*r*) for each nucleotide pair. Relative values of instantaneous *r* should be considered when evaluating these. For simplicity, the sum of *r* values is made equal to 1 for each model. For estimating ML values, a tree topology was automatically computed. Evolutionary analyses were conducted in MEGA 5. Abbreviations used: GTR, General Time Reversible; HKY, Hasegawa–Kishino–Yano; TN93, Tamura–Nei; T92, Tamura three-parameter; K2, Kimura two-parameter; JC, Jukes–Cantor; BIC, Bayesian information criterion; AICc, corrected Akaike information criterion; ln*L*, maximum-likelihood value; G, gamma distribution; I, evolutionarily invariable sites; R, transition/transversion bias; Freq., nucleotide frequencies.

Model	#Param	BIC	AICc	lnL	Invariant	Gamma	R	Freq A	Freq T	Freq C	Freq G
K2+G	33	4246.8	4008.7	-1971.3	n/a	0.20	1.36	0.25	0.25	0.25	0.25
K2+G+I	34	4253.2	4008.0	-1969.9	0.42	0.49	1.40	0.25	0.25	0.25	0.25
T92+G	34	4258.0	4012.8	-1972.3	n/a	0.20	1.35	0.24	0.24	0.26	0.26
T92+G+I	35	4264.5	4012.0	-1970.9	0.42	0.49	1.39	0.24	0.24	0.26	0.26
T92+I	34	4265.6	4020.3	-1976.0	0.68	n/a	1.32	0.24	0.24	0.26	0.26
HKY+G	36	4271.5	4011.8	-1969.8	n/a	0.20	1.37	0.23	0.26	0.24	0.28
TN93+G	37	4274.3	4007.4	-1966.6	n/a	0.21	1.38	0.23	0.26	0.24	0.28
GTR+G	40	4277.7	3989.2	-1954.5	n/a	0.21	1.37	0.23	0.26	0.24	0.28
HKY+G+I	37	4278.0	4011.1	-1968.4	0.42	0.49	1.41	0.23	0.26	0.24	0.28
JC+G	32	4278.1	4047.2	-1991.5	n/a	0.21	0.50	0.25	0.25	0.25	0.25
TN93+G+I	38	4280.7	4006.6	-1965.2	0.41	0.49	1.42	0.23	0.26	0.24	0.28
TN93+I	37	4282.9	4016.0	-1970.9	0.68	n/a	1.33	0.23	0.26	0.24	0.28
GTR+G+I	41	4284.9	3989.2	-1953.4	0.37	0.46	1.39	0.23	0.26	0.24	0.28
JC+G+I	33	4285.5	4047.4	-1990.6	0.39	0.48	0.50	0.25	0.25	0.25	0.25
GTR+I	40	4287.1	3998.6	-1959.1	0.67	n/a	1.34	0.23	0.26	0.24	0.28
K2+I	33	4331.2	4093.1	-2013.5	0.27	n/a	1.18	0.25	0.25	0.25	0.25
JC+I	32	4351.3	4120.4	-2028.1	0.30	n/a	0.50	0.25	0.25	0.25	0.25
K2	32	4367.3	4136.4	-2036.1	n/a	n/a	1.15	0.25	0.25	0.25	0.25
T92	33	4377.4	4139.3	-2036.6	n/a	n/a	1.15	0.24	0.24	0.26	0.26
HKY	35	4391.7	4139.2	-2034.5	n/a	n/a	1.15	0.23	0.26	0.24	0.28
TN93	36	4392.4	4132.7	-2030.2	n/a	n/a	1.16	0.23	0.26	0.24	0.28
JC	31	4393.6	4170.0	-2053.9	n/a	n/a	0.50	0.25	0.25	0.25	0.25
HKY+I	36	4400.9	4141.2	-2034.5	0.00	n/a	1.15	0.23	0.26	0.24	0.28
GTR	39	4407.0	4125.7	-2023.7	n/a	n/a	0.89	0.23	0.26	0.24	0.28

Suppl. Table 2. Maximum-likelihood fits of various nucleotide substitution models for the ITS2 and flanking 5.8S and 28S rDNA locus of *Collyriclum faba*-like specimens. The sequences were trimmed relative to the shortest sequences of the alignment. The analysis involved 49 nucleotide sequences, a total of 565 positions in the final dataset. The sequences of adult *C. faba* from *Saxicola rubetra* (JQ231122) and *Paramacroderoides kinsellai* (HM137665) were used as outgroups. Models with the lowest Bayesian information criterion (BIC) scores are considered to describe the substitution pattern the best. For each model, the corrected Akaike information criterion (AICc) value, maximum likelihood value (ln*L*), and the number of parameters (including branch lengths) are also presented. Non-uniformity of evolutionary rates among sites may be modelled using a discrete Gamma distribution (+G) with five rate categories and by assuming that a certain fraction of sites are evolutionarily invariable (+I). Whenever applicable, estimates of gamma shape parameter and/or the estimated fraction of invariant sites are shown. Assumed or estimated values of transition/transversion bias (*R*) are also shown for each model. They are followed by nucleotide frequencies and rates of base substitutions (*r*) for each nucleotide pair. Relative values of instantaneous *r* should be considered when evaluating these. For simplicity, the sum of *r* values is made equal to 1 for each model. For estimating ML values, a tree topology was automatically computed. Evolutionary analyses were conducted in MEGA 5. Abbreviations used: GTR, General Time Reversible; HKY, Hasegawa–Kishino–Yano; TN93, Tamura–Nei; T92, Tamura three-parameter; K2, Kimura two-parameter; JC, Jukes–Cantor; BIC, Bayesian information criterion; AICc, corrected Akaike information criterion; ln*L*, maximum-likelihood value; G, gamma distribution; I, evolutionarily invariable sites; R, transition/transversion bias; Freq., nucleotide frequencies.

Model	#Param	BIC	AICc	lnL	Invariant	Gamma	R	Freq A	Freq T	Freq C	Freq G
HKY+G	100	5013.1	4186.8	-1993.0	n/a	0.21	1.63	0.23	0.26	0.24	0.27
TN93+G	101	5017.9	4183.3	-1990.3	n/a	0.22	1.63	0.23	0.26	0.24	0.27
HKY+G+I	101	5023.4	4188.8	-1993.0	0.00	0.21	1.63	0.23	0.26	0.24	0.27
TN93+G+I	102	5028.2	4185.4	-1990.3	0.00	0.22	1.63	0.23	0.26	0.24	0.27
K2+I	97	5029.2	4227.6	-2016.5	0.68	n/a	1.54	0.25	0.25	0.25	0.25
HKY+I	100	5047.1	4220.7	-2010.0	0.68	n/a	1.53	0.23	0.26	0.24	0.27
GTR+G	104	5060.1	4200.7	-1996.0	n/a	0.21	1.60	0.23	0.26	0.24	0.27
GTR+G+I	105	5066.7	4199.1	-1994.2	0.43	0.51	1.65	0.23	0.26	0.24	0.27
GTR+I	104	5071.0	4211.7	-2001.5	0.67	n/a	1.54	0.23	0.26	0.24	0.27
JC+I	96	5073.7	4280.4	-2043.9	0.68	n/a	0.50	0.25	0.25	0.25	0.25
HKY	99	5143.4	4325.3	-2063.3	n/a	n/a	1.33	0.23	0.26	0.24	0.27
TN93	100	5145.6	4319.2	-2059.3	n/a	n/a	1.34	0.23	0.26	0.24	0.27
K2	96	5155.2	4361.9	-2084.6	n/a	n/a	1.31	0.25	0.25	0.25	0.25
TN93+I	101	5155.8	4321.2	-2059.3	0.00	n/a	1.34	0.23	0.26	0.24	0.27
T92	97	5166.5	4364.9	-2085.1	n/a	n/a	1.31	0.24	0.24	0.26	0.26
T92+I	98	5176.7	4366.9	-2085.1	0.00	n/a	1.31	0.24	0.24	0.26	0.26
JC	95	5194.6	4409.5	-2109.4	n/a	n/a	0.50	0.25	0.25	0.25	0.25
GTR	103	5205.4	4354.3	-2073.8	n/a	n/a	1.02	0.23	0.26	0.24	0.27
K2+G	97	27771	26969	-13387	n/a	0.20	1.57	0.25	0.25	0.25	0.25
K2+G+I	98	27778	26968	-13385	0.42	0.47	1.63	0.25	0.25	0.25	0.25
T92+G	98	27783	26973	-13388	n/a	0.20	1.56	0.24	0.24	0.26	0.26
T92+G+I	99	27790	26972	-13387	0.42	0.47	1.62	0.24	0.24	0.26	0.26
JC+G	96	27814	27021	-13414	n/a	0.21	0.50	0.25	0.25	0.25	0.25
JC+G+I	97	27822	27021	-13413	0.38	0.47	0.50	0.25	0.25	0.25	0.25

Suppl. Table 3. Maximum-likelihood fits of various nucleotide substitution models for the ITS2 and flanking 5.8S and 28S rDNA locus of Heterophyidae. The sequences were trimmed relative to the shortest sequences of the alignment. The analysis involved 4 nucleotide sequences, a total of 458 positions in the final dataset. The sequences of *Clonorchis sinensis* (KJ137227) and *Euryhelmis costaricensis* (AB521800) were used as outgroups. Models with the lowest Bayesian information criterion (BIC) scores are considered to describe the substitution pattern the best. For each model, the corrected Akaike information criterion (AICc) value, maximum likelihood value (ln*L*), and the number of parameters (including branch lengths) are also presented. Non-uniformity of evolutionary rates among sites may be modelled using a discrete Gamma distribution (+G) with five rate categories and by assuming that a certain fraction of sites are evolutionarily invariable (+I). Whenever applicable, estimates of gamma shape parameter and/or the estimated fraction of invariant sites are shown. Assumed or estimated values of transition/transversion bias (*R*) are also shown for each model. They are followed by nucleotide frequencies and rates of base substitutions (*r*) for each nucleotide pair. Relative values of instantaneous *r* should be considered when evaluating these. For simplicity, the sum of *r* values is made equal to 1 for each model. For estimating ML values, a tree topology was automatically computed. Evolutionary analyses were conducted in MEGA 5. Abbreviations used: GTR, General Time Reversible; HKY, Hasegawa–Kishino–Yano; TN93, Tamura–Nei; T92, Tamura three-parameter; K2, Kimura two-parameter; JC, Jukes–Cantor; BIC, Bayesian information criterion; AICc, corrected Akaike information criterion; ln*L*, maximum-likelihood value; G, gamma distribution; I, evolutionarily invariable sites; R, transition/transversion bias; Freq., nucleotide frequencies.

Model	#Param	BIC	AICc	lnL	Invariant	Gamma	R	Freq A	Freq T	Freq C	Freq G
K2	6	1809.1	1776.0	-882.0	n/a	n/a	1.71	0.25	0.25	0.25	0.25
K2+G	7	1813.3	1774.7	-880.3	n/a	0.21	1.93	0.25	0.25	0.25	0.25
K2+I	7	1815.0	1776.4	-881.2	0.23	n/a	1.73	0.25	0.25	0.25	0.25
JC	5	1816.4	1788.8	-889.4	n/a	n/a	0.50	0.25	0.25	0.25	0.25
T92	7	1816.7	1778.1	-882.0	n/a	n/a	1.71	0.25	0.25	0.25	0.25
K2+G+I	8	1820.8	1776.7	-880.3	0.43	0.65	1.95	0.25	0.25	0.25	0.25
T92+G	8	1820.9	1776.8	-880.4	n/a	0.21	1.93	0.25	0.25	0.25	0.25
HKY	9	1821.3	1771.7	-876.8	n/a	n/a	1.76	0.21	0.28	0.24	0.27
JC+G	6	1821.8	1788.7	-888.3	n/a	0.24	0.50	0.25	0.25	0.25	0.25
JC+I	6	1822.4	1789.3	-888.6	0.44	n/a	0.50	0.25	0.25	0.25	0.25
T92+I	8	1824.1	1780.0	-882.0	0.03	n/a	1.71	0.25	0.25	0.25	0.25
HKY+G	10	1825.9	1770.8	-875.3	n/a	0.19	2.02	0.21	0.28	0.24	0.27
TN93	10	1827.7	1772.6	-876.2	n/a	n/a	1.76	0.21	0.28	0.24	0.27
T92+G+I	9	1828.4	1778.8	-880.4	0.43	0.65	1.94	0.25	0.25	0.25	0.25
HKY+I	10	1828.6	1773.4	-876.6	0.11	n/a	1.76	0.21	0.28	0.24	0.27
JC+G+I	7	1829.3	1790.7	-888.3	0.00	0.24	0.50	0.25	0.25	0.25	0.25
TN93+G	11	1832.6	1771.9	-874.9	n/a	0.21	2.01	0.21	0.28	0.24	0.27
HKY+G+I	11	1833.5	1772.8	-875.3	0.00	0.19	2.02	0.21	0.28	0.24	0.27
TN93+I	11	1835.5	1774.9	-876.4	0.03	n/a	1.76	0.21	0.28	0.24	0.27
TN93+G+I	12	1840.1	1773.9	-874.9	0.00	0.21	2.01	0.21	0.28	0.24	0.27
GTR+G	14	1843.9	1766.7	-869.2	n/a	0.27	1.82	0.21	0.28	0.24	0.27
GTR	13	1844.6	1773.0	-873.4	n/a	n/a	1.32	0.21	0.28	0.24	0.27
GTR+G+I	15	1851.3	1768.7	-869.2	0.41	0.76	1.83	0.21	0.28	0.24	0.27
GTR+I	14	1852.1	1775.0	-873.4	0.14	n/a	1.32	0.21	0.28	0.24	0.27

Suppl. Table 4. Maximum-likelihood fits of various nucleotide substitution models for the ITS2 and flanking 5.8S and 28S rDNA locus of Microphallidae. The sequences were trimmed relative to the shortest sequences of the alignment. The analysis involved 5 nucleotide sequences, a total of 529 positions in the final dataset. The sequence of *Maritrema madrynense* (KF575167) was used as the outgroup. Models with the lowest Bayesian information criterion (BIC) scores are considered to describe the substitution pattern the best. For each model, the corrected Akaike information criterion (AICc) value, maximum likelihood value (ln*L*), and the number of parameters (including branch lengths) are also presented. Non-uniformity of evolutionary rates among sites may be modelled using a discrete Gamma distribution (+G) with five rate categories and by assuming that a certain fraction of sites are evolutionarily invariable (+I). Whenever applicable, estimates of gamma shape parameter and/or the estimated fraction of invariant sites are shown. Assumed or estimated values of transition/transversion bias (*R*) are also shown for each model. They are followed by nucleotide frequencies and rates of base substitutions (*r*) for each nucleotide pair. Relative values of instantaneous *r* should be considered when evaluating these. For simplicity, the sum of *r* values is made equal to 1 for each model. For estimating ML values, a tree topology was automatically computed. Evolutionary analyses were conducted in MEGA 5. Abbreviations used: GTR, General Time Reversible; HKY, Hasegawa–Kishino–Yano; TN93, Tamura–Nei; T92, Tamura three-parameter; K2, Kimura two-parameter; JC, Jukes–Cantor; BIC, Bayesian information criterion; AICc, corrected Akaike information criterion; ln*L*, maximum-likelihood value; G, gamma distribution; I, evolutionarily invariable sites; R, transition/transversion bias; Freq., nucleotide frequencies.

Model	#Param	BIC	AICc	lnL	Invariant	Gamma	R	Freq A	Freq T	Freq C	Freq G
K2	8	2011.9	1964.8	-974.4	n/a	n/a	1.33	0.25	0.25	0.25	0.25
JC	7	2015.0	1973.8	-979.9	n/a	n/a	0.50	0.25	0.25	0.25	0.25
K2+I	9	2019.8	1966.8	-974.4	0.00	n/a	1.33	0.25	0.25	0.25	0.25
K2+G	9	2019.8	1966.8	-974.4	n/a	0.14	1.71	0.25	0.25	0.25	0.25
T92	9	2019.9	1966.9	-974.4	n/a	n/a	1.33	0.25	0.25	0.25	0.25
JC+G	8	2022.9	1975.8	-979.9	n/a	0.15	0.50	0.25	0.25	0.25	0.25
JC+I	8	2022.9	1975.8	-979.9	0.00	n/a	0.50	0.25	0.25	0.25	0.25
HKY	11	2027.1	1962.3	-970.1	n/a	n/a	1.32	0.22	0.28	0.23	0.28
K2+G+I	10	2027.7	1968.8	-974.4	0.00	0.14	1.71	0.25	0.25	0.25	0.25
T92+I	10	2027.8	1968.9	-974.4	0.00	n/a	1.33	0.25	0.25	0.25	0.25
T92+G	10	2027.8	1968.9	-974.4	n/a	0.14	1.71	0.25	0.25	0.25	0.25
JC+G+I	9	2030.8	1977.8	-979.9	0.00	0.15	0.50	0.25	0.25	0.25	0.25
TN93	12	2034.5	1963.9	-969.9	n/a	n/a	1.32	0.22	0.28	0.23	0.28
HKY+I	12	2035.0	1964.4	-970.1	0.00	n/a	1.32	0.22	0.28	0.23	0.28
HKY+G	12	2035.0	1964.4	-970.1	n/a	0.15	1.68	0.22	0.28	0.23	0.28
T92+G+I	11	2035.7	1970.9	-974.4	0.00	0.14	1.71	0.25	0.25	0.25	0.25
TN93+G	13	2042.4	1965.9	-969.9	n/a	0.14	1.73	0.22	0.28	0.23	0.28
TN93+I	13	2042.4	1965.9	-969.9	0.00	n/a	1.32	0.22	0.28	0.23	0.28
HKY+G+I	13	2042.9	1966.4	-970.1	0.01	0.16	1.68	0.22	0.28	0.23	0.28
GTR	15	2049.6	1961.3	-965.6	n/a	n/a	1.05	0.22	0.28	0.23	0.28
TN93+G+I	14	2050.3	1967.9	-969.9	0.00	0.14	1.73	0.22	0.28	0.23	0.28
GTR+G	16	2055.0	1960.8	-964.3	n/a	0.21	1.49	0.22	0.28	0.23	0.28
GTR+I	16	2057.5	1963.3	-965.6	0.00	n/a	1.05	0.22	0.28	0.23	0.28
GTR+G+I	17	2062.8	1962.7	-964.2	0.07	0.41	1.42	0.22	0.28	0.23	0.28

Suppl. Table 5. Maximum-likelihood fits of various nucleotide substitution models for the ITS2 and flanking 5.8S and 28S rDNA locus of Nanophyetidae and Paragonimidae. The sequences were trimmed relative to the shortest sequences of the alignment. The analysis involved 3 nucleotide sequences, a total of 413 positions in the final dataset. The sequence of *Paragonimus kellicotti* (HQ900670) was used as the outgroup. Models with the lowest Bayesian information criterion (BIC) scores are considered to describe the substitution pattern the best. For each model, the corrected Akaike information criterion (AICc) value, maximum likelihood value (ln*L*), and the number of parameters (including branch lengths) are also presented. Non-uniformity of evolutionary rates among sites may be modelled using a discrete Gamma distribution (+G) with five rate categories and by assuming that a certain fraction of sites are evolutionarily invariable (+I). Whenever applicable, estimates of gamma shape parameter and/or the estimated fraction of invariant sites are shown. Assumed or estimated values of transition/transversion bias (*R*) are also shown for each model. They are followed by nucleotide frequencies and rates of base substitutions (*r*) for each nucleotide pair. Relative values of instantaneous *r* should be considered when evaluating these. For simplicity, the sum of *r* values is made equal to 1 for each model. For estimating ML values, a tree topology was automatically computed. Evolutionary analyses were conducted in MEGA 5. Abbreviations used: GTR, General Time Reversible; HKY, Hasegawa–Kishino–Yano; TN93, Tamura–Nei; T92, Tamura three-parameter; K2, Kimura two-parameter; JC, Jukes–Cantor; BIC, Bayesian information criterion; AICc, corrected Akaike information criterion; ln*L*, maximum-likelihood value; G, gamma distribution; I, evolutionarily invariable sites; R, transition/transversion bias; Freq., nucleotide frequencies.

Model	#Param	BIC	AICc	lnL	Invariant	Gamma	R	Freq A	Freq T	Freq C	Freq G
K2	4	1523.5	1503.0	-747.5	n/a	n/a	2.43	0.25	0.25	0.25	0.25
K2+G	5	1529.0	1503.3	-746.6	n/a	0.05	4.28	0.25	0.25	0.25	0.25
K2+I	5	1530.2	1504.5	-747.2	0.25	n/a	2.47	0.25	0.25	0.25	0.25
T92	5	1530.5	1504.8	-747.4	n/a	n/a	2.43	0.24	0.24	0.26	0.26
K2+G+I	6	1533.8	1503.0	-745.4	0.73	0.29	8.05	0.25	0.25	0.25	0.25
JC	3	1534.7	1519.2	-756.6	n/a	n/a	0.50	0.25	0.25	0.25	0.25
T92+G	6	1535.9	1505.1	-746.5	n/a	0.05	4.29	0.24	0.24	0.26	0.26
T92+I	6	1537.5	1506.7	-747.3	0.06	n/a	2.44	0.24	0.24	0.26	0.26
JC+G	4	1539.4	1518.8	-755.4	n/a	0.05	0.50	0.25	0.25	0.25	0.25
HKY	7	1539.8	1503.9	-744.9	n/a	n/a	2.48	0.23	0.25	0.22	0.30
T92+G+I	7	1540.5	1504.6	-745.2	0.73	0.25	10.36	0.24	0.24	0.26	0.26
JC+I	4	1541.8	1521.2	-756.6	0.01	n/a	0.50	0.25	0.25	0.25	0.25
HKY+G	8	1544.6	1503.5	-743.7	n/a	0.05	4.42	0.23	0.25	0.22	0.30
JC+G+I	5	1546.4	1520.7	-755.3	0.26	0.05	0.50	0.25	0.25	0.25	0.25
TN93	8	1546.4	1505.3	-744.6	n/a	n/a	2.49	0.23	0.25	0.22	0.30
HKY+I	8	1546.9	1505.8	-744.9	0.03	n/a	2.49	0.23	0.25	0.22	0.30
HKY+G+I	9	1549.2	1503.0	-742.4	0.73	0.23	11.74	0.23	0.25	0.22	0.30
TN93+G	9	1551.6	1505.4	-743.6	n/a	0.05	4.38	0.23	0.25	0.22	0.30
TN93+I	9	1553.4	1507.2	-744.5	0.05	n/a	2.50	0.23	0.25	0.22	0.30
TN93+G+I	10	1556.2	1504.9	-742.4	0.76	0.28	12.35	0.23	0.25	0.22	0.30
GTR	11	1564.7	1508.3	-743.0	n/a	n/a	2.12	0.23	0.25	0.22	0.30
GTR+G	12	1569.8	1508.2	-742.0	n/a	0.05	3.92	0.23	0.25	0.22	0.30
GTR+I	12	1571.7	1510.2	-743.0	0.09	n/a	2.13	0.23	0.25	0.22	0.30
GTR+G+I	13	1576.9	1510.3	-742.0	0.00	0.05	3.92	0.23	0.25	0.22	0.30

Suppl. Table 6. Maximum-likelihood fits of various nucleotide substitution models for the ITS2 and flanking 5.8S and 28S rDNA locus of Troglotrematidae. The sequences were trimmed relative to the shortest sequences of the alignment. The analysis involved 5 nucleotide sequences, a total of 652 positions in the final dataset. The sequence of Troglotrematidae sp. HS-2009 (AB521803) was used as the outgroup. Models with the lowest Bayesian information criterion (BIC) scores are considered to describe the substitution pattern the best. For each model, the corrected Akaike information criterion (AICc) value, maximumlikelihood value (ln*L*), and the number of parameters (including branch lengths) are also presented. Non-uniformity of evolutionary rates among sites may be modelled using a discrete Gamma distribution (+G) with five rate categories and by assuming that a certain fraction of sites are evolutionarily invariable (+I). Whenever applicable, estimates of gamma shape parameter and/or the estimated fraction of invariant sites are shown. Assumed or estimated values of transition/transversion bias (*R*) are also shown for each model. They are followed by nucleotide frequencies and rates of base substitutions (*r*) for each nucleotide pair. Relative values of instantaneous *r* should be considered when evaluating these. For simplicity, the sum of *r* values is made equal to 1 for each model. For estimating ML values, a tree topology was automatically computed. Evolutionary analyses were conducted in MEGA 5. Abbreviations used: GTR, General Time Reversible; HKY, Hasegawa–Kishino–Yano; TN93, Tamura–Nei; T92, Tamura three-parameter; K2, Kimura two-parameter; JC, Jukes–Cantor; BIC, Bayesian information criterion; AICc, corrected Akaike information criterion; ln*L*, maximum-likelihood value; G, gamma distribution; I, evolutionarily invariable sites; R, transition/transversion bias; Freq., nucleotide frequencies.

Model	#Param	BIC	AICc	lnL	Invariant	Gamma	R	Freq A	Freq T	Freq C	Freq G
K2	8	2349.4	2300.7	-1142.3	n/a	n/a	1.32	0.25	0.25	0.25	0.25
JC	7	2352.1	2309.4	-1147.7	n/a	n/a	0.50	0.25	0.25	0.25	0.25
K2+I	9	2357.5	2302.7	-1142.3	0.00	n/a	1.32	0.25	0.25	0.25	0.25
K2+G	9	2357.5	2302.7	-1142.3	n/a	0.10	1.63	0.25	0.25	0.25	0.25
T92	9	2357.6	2302.8	-1142.4	n/a	n/a	1.32	0.24	0.24	0.26	0.26
JC+G	8	2360.2	2311.5	-1147.7	n/a	0.12	0.50	0.25	0.25	0.25	0.25
JC+I	8	2360.2	2311.5	-1147.7	0.00	n/a	0.50	0.25	0.25	0.25	0.25
K2+G+I	10	2365.6	2304.7	-1142.3	0.00	0.10	1.63	0.25	0.25	0.25	0.25
T92+I	10	2365.7	2304.8	-1142.4	0.00	n/a	1.32	0.24	0.24	0.26	0.26
T92+G	10	2365.7	2304.8	-1142.4	n/a	0.10	1.63	0.24	0.24	0.26	0.26
HKY	11	2368.1	2301.1	-1139.5	n/a	n/a	1.32	0.22	0.26	0.23	0.28
JC+G+I	9	2368.3	2313.5	-1147.7	0.00	0.12	0.50	0.25	0.25	0.25	0.25
T92+G+I	11	2373.8	2306.8	-1142.4	0.00	0.10	1.63	0.24	0.24	0.26	0.26
TN93	12	2376.0	2302.9	-1139.4	n/a	n/a	1.32	0.22	0.26	0.23	0.28
HKY+G	12	2376.2	2303.1	-1139.5	n/a	0.09	1.65	0.22	0.26	0.23	0.28
HKY+I	12	2376.2	2303.1	-1139.5	0.00	n/a	1.32	0.22	0.26	0.23	0.28
TN93+I	13	2384.1	2304.9	-1139.4	0.00	n/a	1.32	0.22	0.26	0.23	0.28
TN93+G	13	2384.1	2304.9	-1139.4	n/a	0.10	1.64	0.22	0.26	0.23	0.28
HKY+G+I	13	2384.3	2305.1	-1139.5	0.00	0.09	1.65	0.22	0.26	0.23	0.28
GTR	15	2391.7	2300.4	-1135.1	n/a	n/a	1.12	0.22	0.26	0.23	0.28
TN93+G+I	14	2392.2	2306.9	-1139.4	0.00	0.10	1.64	0.22	0.26	0.23	0.28
GTR+G	16	2398.6	2301.1	-1134.5	n/a	0.29	1.43	0.22	0.26	0.23	0.28
GTR+I	16	2399.8	2302.4	-1135.1	0.00	n/a	1.12	0.22	0.26	0.23	0.28
GTR+G+I	17	2406.7	2303.2	-1134.5	0.11	0.38	1.43	0.22	0.26	0.23	0.28
